# Dealing with Illumination in Visual Scenes: Effects of Ageing and Alzheimer's Disease

**DOI:** 10.1371/journal.pone.0045104

**Published:** 2012-09-24

**Authors:** Gillian Porter, Ute Leonards, Tom Troscianko, Judy Haworth, Antony Bayer, Andrea Tales

**Affiliations:** 1 Department of Experimental Psychology, University of Bristol, Bristol, United Kingdom; 2 South Glos Memory Service, Avon and Wiltshire Mental Health Partnership, Bristol, United Kingdom; 3 Department of Geriatric Medicine, Cardiff University, Cardiff, United Kingdom; Oregon Health & Science University, United States of America

## Abstract

Various visual functions decline in ageing and even more so in patients with Alzheimer's disease (AD). Here we investigated whether the complex visual processes involved in ignoring illumination-related variability (specifically, cast shadows) in visual scenes may also be compromised. Participants searched for a discrepant target among items which appeared as posts with shadows cast by light-from-above when upright, but as angled objects when inverted. As in earlier reports, young participants gave slower responses with upright than inverted displays when the shadow-like part was dark but not white (control condition). This is consistent with visual processing mechanisms making shadows difficult to perceive, presumably to assist object recognition under varied illumination. Contrary to predictions, this interaction of “shadow” colour with item orientation was maintained in healthy older and AD groups. Thus, the processing mechanisms which assist complex light-independent object identification appear to be robust to the effects of both ageing and AD. Importantly, this means that the complexity of a function does not necessarily determine its vulnerability to age- or AD-related decline.

We also report slower responses to dark than light “shadows” of either orientation in both ageing and AD, in keeping with increasing light scatter in the ageing eye. Rather curiously, AD patients showed further slowed responses to “shadows” of either colour at the bottom than the top of items as if they applied shadow-specific rules to non-shadow conditions. This suggests that in AD, shadow-processing mechanisms, while preserved, might be applied in a less selective way.

## Introduction

A wealth of literature shows that both healthy ageing and Alzheimer's disease are associated with accompanying decrements in sensory and cognitive performance. Moreover, there is an assumption that functions which involve more complex processing are more likely to be vulnerable, presumably because age-related decline accumulates across multiple-component processes, or because compensation mechanisms become less effective as complexity increases, e.g. [1].

One function which is considered particularly complex is the ability to distinguish “material” from “light” within the visual input [2]; that is, to distinguish “real objects” from shadows. A given physical object is associated with very different retinal input depending upon the strength and direction of lighting, and whether or not the illumination is occluded by other items to create cast shadows. Successful object recognition requires, in part, that the brain can ignore any light-related variability in the visual input, while simultaneously processing the variability (such as texture) arising from the material characteristics of the surfaces present. Despite decades of research on the subject, it remains unclear exactly how this distinction is made [2]. A frequent assumption is that colour vision evolved in order to solve this kind of problem – colour is constant across many illumination changes [3]. However, the visual system can be confronted with scenes largely devoid of colour information, and then faces a major challenge in computing surface information. It seems likely that numerous complex higher-level heuristics are used for identifying light-related variability [2], [4], and these determine the extent to which basic lower-level features of the input are accessible, with the features relating to object properties being the most comprehensively processed. The underlying mechanisms are likely to be complicated and to involve information circulating between multiple cortical levels [5]. In this paper we examine whether these complex mechanisms appear to be disrupted by cognitively healthy ageing and AD.

Evidence is emerging that some of the more complex aspects of visual processing, including those contributing to object recognition, may become disrupted by pathological and even by healthy ageing. The optical properties of the eye change as people get older, resulting in alterations to basic visual sensitivities [6]–[9], together with some of the processes on which these depend, e.g. [10]. In addition, recent reports indicate that older people perform less well than the young at integrating certain types of basic visual information over time or space to identify structure [11]–[13]. One crucial factor to the identification of structure is to correctly distinguish lighting from material, and there is evidence in various neurological conditions that this specific ability can be disrupted, causing difficulties with object recognition. Becchio et al [14], for example, found cast shadow information to interfere with object recognition for autistic children, but assist performance in typically developing children. Similarly, visual neglect can disrupt shadow processing [15]. While not suggesting any parallels between those neurological dysfunctions and ageing or AD, we wondered whether ageing might also reduce the ability to appropriately process lighting-related visual information? This has been little investigated, although Norman & Wiesemann [16] found judgements of surface orientation from shading and highlights to be age-impaired in one experiment and age-equivalent in another. Here we focus on cast shadows: the failure to discard these as lighting “artefacts” by misinterpreting them as objects or parts of objects might increase the visual clutter present in a scene. Such a suggestion would fit well with observations that sensitivity to visual clutter increases with age [17]–[18].

Visual changes are more marked in patients with Alzheimer's disease (AD) than in normal ageing, and span a wide range of visual [19] and attention-related tasks; for review see [20]. Changes include significantly greater impairments than healthy people of the same age in integrating spatial information to identify form [21], and in certain types of inhibitory processes [22]. Inhibitory mechanisms are often assumed to be crucial to ignoring lighting-related variability in the visual input, e.g. [23]. Also, we know that other types of brain dysfunction can disrupt shadow processing as stressed above [14]–[15]. Moreover, anecdotally, some AD patients experience considerable confusion relating to clutter in the visual environment. We therefore asked whether AD might particularly disrupt the complex processing involved in identifying and correctly classifying “lighting” in the visual input.

**Figure 1:**
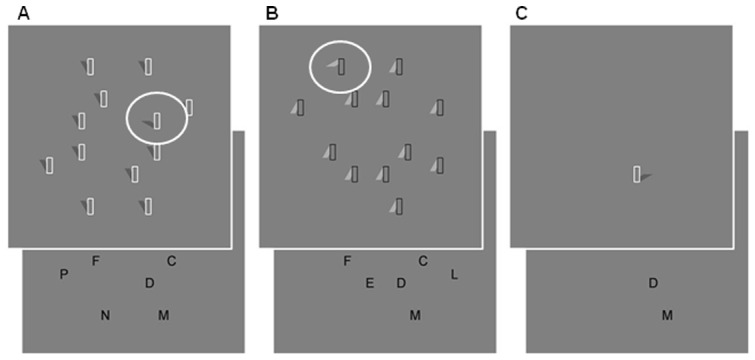
Example stimulus displays for a) the search task with dark shadows, upright; b) the search task with white non-shadows, inverted; c) the detection task. Each trial began with presentation of a central fixation cross, for a fixed 1 s (a/b) or a variable 1–2 s (c). Displays were then presented until the participant keypress indicating target identification (a/b) or item detection (c). The subsequent display of letters allowed the target location to be reported.

A methodology which reliably demonstrates categorization of lighting-related visual information was described by Rensink and Cavanagh [23]. Using displays of items (see Figure 1a) interpretable as posts with shadows cast by light-from-above, as in natural lighting, they found slower visual search for a discrepant “shadow” than when searching exactly the same displays inverted, when presumably the items are not seen as shadows because this interpretation is inconsistent with light-from-above. Thus, the visual system has more difficulty in identifying the shape of shadow-like (lighting-related) images than of equivalent images which are not interpreted as shadows and are therefore object-related; see also [5]. Here we used these same stimuli to test the hypothesis that the separation of “lighting” from “material” might be impaired in ageing and especially AD, resulting in lighting-related information being more visible to these older groups. If so, the differences between searching for shadow-like items and their inverted controls should disappear.

The data reported below show some effects specific to ageing and Alzheimer's disease, but both our older and AD sample groups maintained clear evidence of slowed search among shadow-like stimuli (dark regions at the bottom of items), relative to stimuli which were less shadow-like. This fails to support our hypothesis that older people may find shadows more visible (and thus more confusing) than the young; if anything, the data as a whole suggest the contrary: information from shadows seems even more inaccessible. Thus, despite the complexity of the mechanisms likely to be involved, the “suppression” of cast shadows within visual input appears to survive the effects of ageing and AD. This has implications for assumptions that more complex processes are inevitably more vulnerable to the detrimental effects of ageing.

## Methods

### Ethics Statement

The study was conducted according to the principles in the Declaration of Helsinki. It was approved by Frenchay and South East Wales Research Ethics Committees and all participants gave written informed consent to participate.

Only people with the capacity to consent were included in the study (in keeping with the requirements of the ethics committee). Assent from family or carers was not sought. Capacity was assessed by clinicians (AB and JH) with specialist expertise in this field and consistent with the requirements of the Mental Capacity Act. Thus all the people included had the capacity to understand, retain and weigh up information about the study and had then independently communicated their interest in taking part and provided their written consent.

All consent procedures were approved by the two relevant ethical review boards.

### Participants

Details of the older participants are provided in Table 1. The patient group (n = 13) was recruited from Bristol and Cardiff memory clinics. All had a recent diagnosis of probable AD according to current guidelines (*DSM-IV* and *NINDS–ADRDA*), with Mini-Mental State Examination (*MMSE*) [24] scores, indicating severity of dysfunction, of 16–26. Those in the upper range represented a significant decline from a previously higher level of functioning (as indicated by years of education). Four patients were stable on anti-cholinesterase drugs while the remainder were taking no medication likely to affect cognitive performance. A control group of 18 older adults was recruited via the same memory clinics, although two were excluded from analysis due to red/green colour vision deficiency. Those tested in Bristol had been assessed as being cognitively healthy by the recruiting clinician (JH) via recent administration of a full neuropsychological test battery. The Cardiff recruits were spouses of patient participants, judged by the recruiting clinician (AB) to be unimpaired, and this was checked via administration of the MMSE and National Adult Reading Test (NART) at the time of testing. A young control group of 18 participants aged 18–27 years was recruited from the University of Bristol. All participants corrected their vision as necessary using their own spectacles or contact lenses. For the older groups, adequate corrected visual acuity for the task was ascertained from the ability to read standard newsprint at arm's length, and contrast sensitivity using the Functional Acuity Contrast Test (FACT; Stereo Optical Company Inc). Ability to see the stimuli sufficiently well to perform the required judgements was confirmed when explaining the task.

**Table 1:** Clinical and demographic details for all older participants.

	Group	Location	Sex	Age	AchEIs	MMSE	IQ	Years Ed	FACT
1	AD	Cardiff	M	78	No	19	89	11	40
2	AD	Cardiff	M	77	Yes	26	123	18	40
3	AD	Cardiff	M	83	No	22	117	9	100
4	AD	Cardiff	M	67	Yes	26	123	17	25
5	AD	Cardiff	M	67	Yes	24	118	15	30
6	AD	Bristol	M	75	No	19	103	9	80
7	AD	Bristol	M	82	No	16	112*	13	40
8	AD	Bristol	F	88	No	24	112	9	30
9	AD	Bristol	M	77	No	21	101	9	80
10	AD	Bristol	F	77	No	22	112*	13	30
11	AD	Bristol	F	78	No	24	103	9	30
12	AD	Bristol	M	69	Yes	26	116*	15	40
13	AD	Bristol	M	74	No	23	95	9	25
**Mean**	**AD**		**10 M**	**76.3**		**22.5**	**110**	**12.0**	**45.4**
1	Older	Cardiff	F	75	-	30	122	17	25
2	Older	Cardiff	F	78	-	28	116	9	30
3	Older	Cardiff	F	62	-	30	115	11	25
4	Older	Bristol	M	81	-	27	103	13	30
5	Older	Bristol	M	75	-	27	100	19	30
6	Older	Bristol	F	77	-	28	123	11	25
7	Older	Bristol	F	74	-	27	118	15	25
8	Older	Bristol	M	80	-	26	121	13	40
9	Older	Bristol	F	69	-	25	123	16	25
10	Older	Bristol	M	73	-	28	122	11	25
11	Older	Bristol	M	74	-	29	107	11	30
12	Older	Bristol	M	64	-	26	121	12	20
13	Older	Bristol	M	79	-	28	119	11	40
14	Older	Bristol	F	80	-	27	126	14	25
15	Older	Bristol	M	76	-	28	123	12	30
16	Older	Bristol	M	84	-	28	124	14	80
**Mean**	**Older**		**9 M**	**75.3**	**-**	**27.6**	**118**	**13.0**	**31.6**
**Mean**	**Young**	**Bristol**	**9 M**	**20.1**	**-**	**-**	**-**	**15.7**	**-**

**Legend:**

AchEIs – denotes whether or not patients were taking acetylcholinesterase inhibitors.

MMSE – Mini Mental State Examination score (maximum 30).

IQ – estimated from errors in the National Adult Reading Test, or * demographic factors.

Years Ed – number of years of full time education.

FACT – score on the Functional Acuity Contrast Test, expressed as x/20.

Chi-squared analysis found no significant difference between any of the three groups in terms of sex (p>0.01). Independent t-tests showed that the two older groups did not differ from each other in terms of age (p = 0.662) or years' education (p = 0.771), but the young group were significantly younger (both p<0.001) and more educated than the older (p = 0.001) and AD (p = 0.002) groups. In keeping with the group definitions, MMSE was significantly lower in the AD group than among older controls (t(15.8) = 5.63, p<0.001; corrected for unequal variances). Measures of IQ suggested slightly higher intelligence in the older controls than premorbidly in the AD group (t(27) = 2.39, p = 0.024). Eyesight (contrast sensitivity), measured by the FACT, was weaker for the AD group than the older controls but not significantly so (p = 0.069).

### Stimuli and tasks

The main experiment involved a target-present visual search task whereby participants were asked to search for a single target, always present within the display, and press the spacebar as soon as it was located. Reaction time (RT) was recorded from this keypress. All search items then disappeared and each was immediately replaced in the same on-screen location by a single unique upper-case letter. The participant was required to speak aloud the letter corresponding to the target location, for recording and later accuracy-checking by the experimenter. This approach, as used previously by Eglin et al. [25], was designed to minimise noise in the data due to difficulties in remembering which key represented target-present and which target-absent responses.

Stimulus elements were based upon those used by Rensink & Cavanagh [23] and consisted of a rectangular shadow caster, 12 mm tall by 4 mm wide, defined by a 0.6 mm outline, with an oblique solid “shadow” attached, apparently behind the shadow caster. Distractor elements were all identical, with the “shadow” oriented at 30° to the vertical, while the target element (one per display) had the “shadow” oriented at 60° to the vertical. The area of the “shadow” was equated across target and distractor elements. The *dark shadow* test condition (Figure 1a) involved shadows of measured luminance 7.74 cd/m^2^ against a background of 13.2 cd/m^2^, with white shadow casters (124 cd/m^2^). In the *white non-shadow* control condition (Figure 1b), shadows were 23.3 cd/m^2^ with black shadow casters (0.363 cd/m^2^) against the same background. The central fixation cross measured 4 mm across and was of 0.363 cd/m^2^ luminance.

Displays included either 6 or 12 elements. Each appeared in one of 16 possible locations forming two concentric rings of eight, evenly spaced around the centre of the screen, of diameters 63 mm and 117 mm. Locations were randomly chosen on each trial within the constraints that items were always equally split between inner and outer rings and the target occurred equally often in the inner and the outer ring. Items could be presented upright or inverted and with shadows to the left or the right of the shadow caster, but this was consistent within each display.

In addition, to measure basic response speeds participants completed a detection task using the same stimuli. Each trial involved only a single target element appearing in one of the 16 locations. In this case, the task was to press the spacebar immediately on seeing the item, giving an RT measure, and then report which of six letter locations in the subsequent display corresponded to the item location (see Figure 1c). Each location was used equally often in this task.

### Procedure

Older participants were tested in a memory clinic setting with the lights off and curtains closed. Younger participants were tested in a windowless laboratory with lights dimmed. Displays were presented using Matlab and the Psychophysics Toolbox [26] on a Dell Precision M4300 15.5″ laptop computer, viewed at a comfortable distance, other than three older participants (two in the AD group) who were tested on a Toshiba Tecra M4 laptop using the same stimulus dimensions and luminances. The spacebar was clearly marked with a coloured sticker and was used both to initiate each trial and for response. One block of 36 trials of the detection task was completed first. Since shadow discrimination was not required, the shadow conditions were intermixed. Data from the first four trials were discarded, which gave the experimenter the opportunity to explain the task fully with reference to example stimuli, and the participant the chance to practice, before trials proper began. Each trial involved the fixation display presented for a variable period of between 1 and 2 s, then the detection display, without fixation, until response. The 32 subsequent trials included two of each combination of shadow colour (dark/white), orientation (upright/inverted), shadow side (left/right) and eccentricity (inner/outer), presented in randomised order.

The search task was then introduced with a practice block of 20 trials of the first shadow condition. Shadows were not mentioned at any stage when describing the task; the stimuli were explained in terms of discrepant angles, although participants themselves often referred to them as shadows. Each trial began with presentation of the fixation cross for 1 s, then the search display (without fixation) until response. Four test blocks followed, each of 36 trials, with shadow conditions presented separately in the order ABBA or BAAB, counterbalanced within each sample group. The first four trials of each block were discarded, again to allow participants to be reminded of the task. The following 32 trials proper comprised two of each combination of orientation (upright/inverted), set size (6/12), shadow side (left/right), and target eccentricity (inner/outer), in randomised order. Finally, a second block of the detection task was completed, exactly like the first.

### Analysis

Trials classified as anticipations (reaction time <80 ms, so representing an accidental keypress) were excluded. These occurred more frequently with older participants but did not exceed 11 trials in total (5% of all trials) for any individual and did not differ in frequency between healthy older participants and ADs. The percentage of omissions or errors within the remainder was calculated to gauge response accuracy. All participants performed each version of the search task with over 80% accuracy. Median RTs were then calculated across correctly answered trials for each individual for each combination of shadow type, orientation and set size. Each participant's median single-stimulus detection RT (sRT), calculated across all conditions, was then used to control for marked individual and group variation in overall response speeds by recalculating search RTs as (RT–sRT)/sRT.

These normalised RTs were those entered into Analysis of Variance, with shadow colour (2), orientation (2) and set size (2) as repeated measures. Significant effects were explored using Tukey *post-hoc* tests. The key result with regard to our hypothesis was the shadow colour by orientation interaction. This was expected to be present for the younger group, with a significant orientation effect for dark shadows but not white non-shadows, but we predicted that the interaction would be absent in the older and/or AD groups. Initial analyses included test order (dark or white shadow first) as a variable, but this was omitted once shown to have no effect. For simplicity, we do not report any interactions including set size as a moderator since it is irrelevant to the hypothesis presented here.

## Results

**Figure 2:**
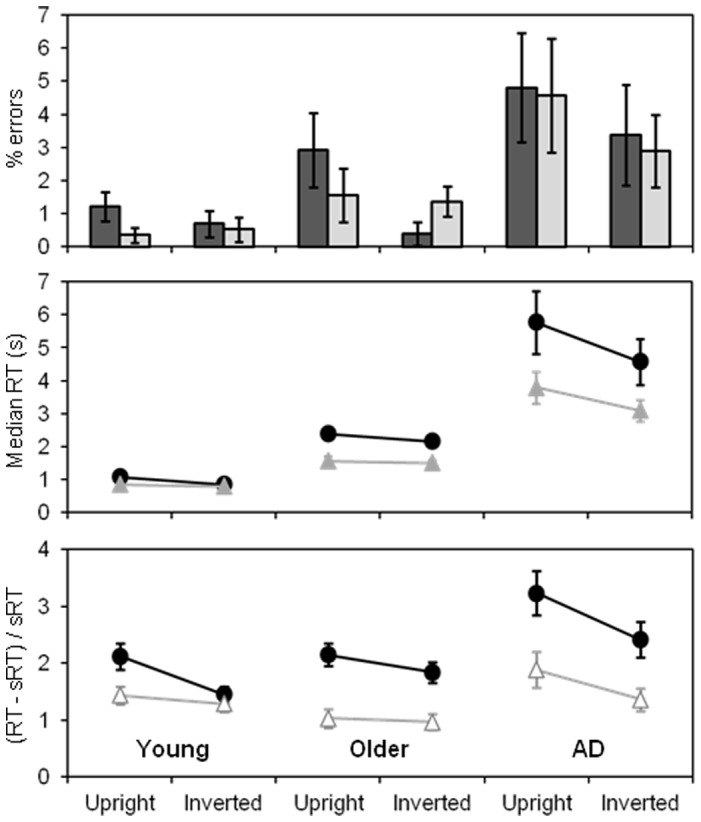
Performance data for each sample group in the search task, showing interaction of shadow type with item orientation. Upper panel shows errors, middle panel shows raw median RTs and lower panel shows RTs normalised according to single-stimulus RTs. Dark bars/circles represent dark shadows and light bars/triangles, white non-shadows. Error bars represent ±1 SEM.

Figure 2 shows the error and reaction time data from the search task for each group, separated by test condition (shadow vs white non-shadow and upright vs inverted). In the middle panel, the raw RTs show an overall slowing from young to older and then to AD participants, accompanied by an increase in RT variability. From the lower panel, depicting the RTs normalised to single item detection speeds as described above, it can be seen that normalisation considerably reduced differences in RT and RT variability between the three groups as intended.

An initial ANOVA included all three participant groups, using the normalised data and with the different test conditions as repeated measures. We describe first the general search patterns emerging from this analysis before focusing on the shadow colour×orientation interaction which is central to our hypothesis.

This overall ANOVA showed main effects of shadow colour (F(1, 44) = 67.7, MSE = 1.04, p<0.001, η^2^_p_ = 0.606; RTs to stimuli with dark “shadows” slower than white), item orientation (F(1, 44) = 58.1, MSE = 0.285, p<0.001, η^2^_p_ = 0.569; RTs to upright stimuli slower than inverted) and group (F(2, 44) = 4.10, MSE = 4.36, p = 0.023, η^2^_p_ = 0.157). *Post-hoc* Tukey analysis revealed that, after accounting for generalised slowing through normalisation of the data, AD patients remained disproportionately slower than healthy old (p = 0.030) and, marginally, young groups (p = 0.052), but young and healthy old RTs did not differ. Group and shadow colour interacted significantly (F(2, 44) = 5.05, MSE = 1.04, p = 0.011, η^2^_p_ = 0.187), with both older groups (p<0.001, Tukey), but not the young (p = 0.153), showing slower responses to dark than white-shadow stimuli. Group also interacted with item orientation (F(2, 44) = 5.92, MSE = 0.285, p = 0.005, η^2^_p_ = 0.212), with young and AD groups (both p<0.001) but not healthy old (p = 0.359), showing slower responses to upright than inverted items. This unusual pattern, of similarity between young and AD but not old groups, will be considered in the discussion.

The important shadow colour×orientation interaction was significant overall (F(1, 44) = 12.7, MSE = 0.226, p = 0.001; η^2^_p_ = 0.224), although upright RTs were slower than inverted for both dark (p<0.001, Tukey) and white (p = 0.005) shadow-like stimuli. Contrary to our expectations, however, the 3-way group×shadow colour×orientation interaction was non-significant, indicating that all groups showed a similar behaviour overall with respect to shadow processing (p = 0.435; lower panel, figure 2). Given our specific predictions relating to this 3-way interaction, *post-hoc* Tukey tests were used to explore the shadow colour×orientation effect in each participant group separately. For the young group, the data conformed to previous reports, showing an orientation effect for dark (p<0.001) but not white (p = 0.973) stimuli. The pattern was less clear in the older groups. For the healthy older group, though going in the same direction as in young participants, the orientation effect was non-significant for both stimulus colours (dark p = 0.307, white p = 1.00). For the AD group, orientation moderated RTs for both dark (p<0.001) and white (p = 0.011) stimuli again in the same direction as in young participants.

We repeated this analysis with a separate ANOVA for each sample group (repeated measures across the test conditions in each case), in order to exclude any potential difficulties of unequal variance between groups which had persisted even after normalisation of individual data. In young participants, the shadow colour×orientation interaction was again confirmed (F(1, 17) = 13.2, MSE = 0.187, p = 0.002; η^2^_p_ = 0.437) with an orientation effect for dark shadows (p<0.001) but not white (p = 0.493). For healthy older participants taken alone, the key interaction approached significance (F(1, 15) = 4.24, MSE = 0.110, p = 0.057; η^2^_p_ = 0.220) and *post-hoc* analyses showed the orientation effect for dark (p = 0.010, Tukey) but not white shadow stimuli (p = 0.844); thus, patterns were overall the same as in young participants though less pronounced. For the AD group, the overall interaction was non-significant (p = 0.275) but nevertheless RTs to dark shadows were significantly affected by orientation (p = 0.003, Tukey) while those to white shadows were only marginally so (p = 0.054). Identical patterns were found for raw (non-normalised) RTs analysed separately by group (middle panel, figure 2).

## Discussion

In this study, we looked at a visual task often considered complex and difficult - deciding what is an illumination change (a shadow) and what is a material change (an object). Recent literature has looked at people's ability to solve such problems, and found that successful solutions depend on integrating several assumptions about light and objects - in particular, that light comes from above and that shadows are dark rather than light, e.g. [2]–[5].

Using a paradigm which has been well tested in the recent literature [23], we investigated the functioning of healthy older people and Alzheimer's patients on this complex task - and found no deficits in the associated visual processing. As explained below, the results here indicate that light-related variability in the form of shadows might become less visible in ageing & AD, rather than more visible as we originally suggested. However, any such changes appear not to arise from changes to lighting-specific processing mechanisms. Our data suggest that in older people and AD patients, such mechanisms (for cast shadows at least) appear robust, and such people efficiently utilise the same assumptions about the behaviour of light as normal young observers.

### Orientation specific shadow processing

If lighting-related visual information is correctly classified as such and discounted, then the dark-shadow stimuli should be less easily distinguished when upright (shadow-like) than when inverted (not shadow-like), leading to longer upright than inverted RTs. The same shapes coloured white, and hence unlike shadows, would not be expected to show this orientation-related RT difference. Based on previous reports [5], [23], [27]–[28] we expected this interaction to be apparent in our younger participants, but we hypothesised that it would be reduced or absent in the older groups. The overall ANOVA across all three participant groups showed the expected shadow colour×orientation interaction but, contrary to our predictions, the pattern was not moderated by sample group. This provides a first indication that processing which distinguishes “lighting” from “material” may not be greatly altered by either healthy ageing or AD.

This was further confirmed by separate analyses within each sample group, examining the shadow colour×orientation relationship: in all three participant groups, upright dark shadow-like image regions were less easily perceived than equivalent images which were inverted and so not perceived as shadow-like. The pattern is sufficiently robust to remain apparent within the AD group, despite the greater variability between individuals. Although the pattern was cleanest for the younger group, we found no real evidence that the effect was abolished in either healthy older or AD samples.

These data thus support the view that the perceptual operations which allow us to classify images and discount variability within them induced by lighting remain largely intact in both healthy ageing and AD, at least with respect to shadow processing. While this is compatible with previous conclusions [16], it is surprising given the complex heuristics assumed to make such classifications [2], [4] and the types of visual processing for which there is evidence of age-related decline. Ageing is typically thought to disrupt complex processes more than simple ones: older people maintain the ability to discriminate relatively simple shapes or features, including orientation [10]–[11], [13] while showing impaired integration of features or shapes across time or space [11]–[13], [29]. Complex task deficits are also greater than simpler task deficits in AD [21], [30]. However, the identification and ignoring of lighting-related variability involves complex, multi-stage processing [5], yet here we see the mechanisms continue to function even in AD. This tends to oppose such complexity theories. Similarly, if inhibitory mechanisms are involved in shadow-related processing as suggested by Rensink & Cavanagh [23], the present data show no evidence of the decline of inhibition usually associated with older age [22], [31]–[34].

Instead, the present data support the idea that visual perception-related decline in ageing and AD is specific to certain processes, rather than a general phenomenon. If so, one might expect that, for example, age-related difficulties with contour integration could arise specifically from impaired neural synchronisation or binding processes across cortico-cortical connections [13]; see also [35]. In particular, if long-range cortico-cortical connections between different brain areas were required, then AD patients with occipital white matter atrophy should show exaggerated impairments, as found by Ulhaas et al [21]. The absence of impairments in the present data would then suggest that shadow-processing mechanisms do not depend on such cortico-cortical connections, neuronal synchronisation and binding. Instead, the mechanisms involved in separating light from material, although complex, might depend upon the more automatic types of processing that are more typically spared in AD [20], [22]; but see [36].

### Other issues affecting shadow visibility

Although the shadow×orientation interaction data suggest that processing difficulties specific to upright shadows remain intact in ageing and AD, there was evidence of two other group-specific perceptual differences (their co-occurrence weakening the statistical effect of the key shadow×orientation interaction in the older groups). Firstly, both older groups, but not the young, showed far slower responses to stimuli with dark than white shadow-like regions regardless of item orientation (note the differing vertical separation of the lines in figure 2). This is likely to reflect increasing light scatter in the ageing eye, resulting in increasingly greater difficulty in perceiving dark stimuli on a lighter background than vice versa [37]–[38]. Thus, shadows (i.e. regions darker than the background on which they fall) are likely to be less readily apparent to older than younger people for purely optical reasons.

Secondly, for the AD group, responses to upright items with “shadows” at the bottom were significantly slower than responses to inverted items with “shadows” at the top, regardless of shadow colour (note the steeper slope of both lines for the AD group in figure 2). This is in clear contrast to the young participants, in which such an orientation difference was restricted to darker stimuli only. This orientation effect, specific to the AD group, is curious and has not, to our knowledge, been previously reported. It almost seems as if AD patients were using the heuristics of “light from above” with shadows at the bottom in a non-selective way; i.e. outside the context of the direction of contrast polarity, considering only dark regions as shadows, not lighter regions. Here we can only speculate why this might be the case. This effect may relate to reported lower visual field deficits in AD patients [39]–[41], perhaps causing lower stimulus regions to be less readily perceived than upper regions. Alternatively, this might hint towards an attentional dysfunction affecting the processing of the lower parts of objects within an object-centred reference frame similar to that which has been described for object-centred visual neglect, e.g. [42]. Future studies will have to investigate this effect further, especially as a distinction between shadows and objects might be of particular importance in both the lower visual field and the lower part of an object to enable safe locomotion and identification of obstacles. If so, such an effect may mean that shadows falling below their shadow-casters are less visible to AD patients than healthy people, due to specific visual processing biases.

### General comments

Could these results be explained by demographic or other differences between the groups? The younger participants had received more education than the older people tested, and the AD group was seemingly of slightly lower pre-morbid intelligence than the healthy older group. We cannot entirely dismiss the contribution of these factors, but if they influenced the perceptual judgements being examined here, we would expect this to result in differences between the groups, rather than the similarities on which we focus. Possibly, weaker visual acuity in the AD group than controls (albeit non-significant) may have contributed to the AD-specific difficulties with upright non-shadow stimuli, but the contrast sensitivity differences seem insufficiently large to fully drive this effect.

Note, however, that this is a small scale study, and focusing on a very specific type of judgement. Only with converging evidence from further studies, using different methodologies, will the extent to which the processes for separating “light” from “material” are impervious to ageing and dementia be clear.

In a wider context, these results imply that the fact that a task requires complex assumptions and processing does not necessarily mean that the task will be more difficult for older people or AD patients. Whether a task is associated with a deficit may be governed by other issues, such as the difficulty of comparing neural activity across larger cortical distances, or pairing up sets of information which may be degraded. Some complex tasks survive these problems and here we present an important example of one such task. Our understanding of what happens to the brain in old age and in dementia may have to be revised to take account of the idea that task complexity does not predict the degree of cognitive deficits.
